# 3D Point Cloud Acquisition and Correction in Radioactive and Underwater Environments Using Industrial 3D Scanners

**DOI:** 10.3390/s22239053

**Published:** 2022-11-22

**Authors:** Dongjun Hyun, Sungmoon Joo, Ikjune Kim, Jonghwan Lee

**Affiliations:** Korea Atomic Energy Research Institute, 989-111 Daedeok-daero, Yuseong-gu, Daejeon 305-353, Republic of Korea

**Keywords:** 3D scanner, remote dismantling system, radiation protection, refraction correction, underwater

## Abstract

This study proposes a method to acquire an accurate 3D point cloud in radioactive and underwater environments using industrial 3D scanners. Applications of robotic systems at nuclear facility dismantling require 3D imaging equipment for localization of target structures in radioactive and underwater environments. The use of industrial 3D scanners may be a better option than developing prototypes for researchers with basic knowledge. However, such industrial 3D scanners are designed to operate in normal environments and cannot be used in radioactive and underwater environments. Modifications to environmental obstacles also suffer from hidden technical details of industrial 3D scanners. This study shows how 3D imaging equipment based on the industrial 3D scanner satisfies the requirements of the remote dismantling system, using a robotic system despite insufficient environmental resistance and hidden technical details of industrial 3D scanners. A housing unit is designed for waterproofing and radiation protection using windows, mirrors and shielding. Shielding protects the industrial 3D scanner from radiation damage. Mirrors reflect the light required for 3D scanning because shielding blocks the light. Windows in the waterproof housing also transmit the light required for 3D scanning with the industrial 3D scanner. The basic shielding thickness calculation method through the experimental method is described, including the analysis of the experimental results. The method for refraction correction through refraction modeling, measurement experiments and parameter studies are described. The developed 3D imaging equipment successfully satisfies the requirements of the remote dismantling system: waterproof, radiation resistance of 1 kGy and positional accuracy within 1 mm. The proposed method is expected to provide researchers with an easy approach to 3D scanning in radioactive and underwater environments.

## 1. Introduction

In recent years, many industrial 3D scanners have been introduced that have achieved sufficient performance to meet the needs of various industries and research fields. However, most industrial 3D scanners are designed to work in normal environments, and some special purpose 3D scanners are very expensive and do not support a wide range of applications. When a researcher develops a 3D scanner suitable for research purposes using well-known basic knowledge, the developed 3D scanner may show insufficient performance compared to industrial 3D scanners, which have competitiveness in the market.

This study starts from the need of 3D imaging equipment for a remote dismantling system using a robotic system in nuclear facility dismantling. When dismantling a nuclear facility that has been in operation for several decades, highly activated structures must be remotely dismantled underwater to prevent exposure to equipment and workers. Existing nuclear facility decommissioning projects have been carried out in a way that large, submerged cutting equipment is operated by field workers above the water surface. Since long-term low-dose exposure to workers cannot be avoided despite the protection of water and all simple repetitive tasks are performed manually, a remote dismantling system using robots is required for safety and efficiency. The 3D imaging equipment is necessary for localization of a target structure of a robotic system in radioactive and underwater environments.

In the remote dismantling system developed by us, the robotic system uses a pre-planned path created by a digital manufacturing system developed in our previous research [1]. Since the pre-planned path is created in a virtual environment, aligning the pre-planned path to the actual target structure is needed through target localization using 3D scanning. The proposed remote dismantling system implements a semi-automated robotic system through the pre-planned path and target localization instead of the direct teleoperation or shared teleoperation in previous studies [2,3,4]. Since both direct teleoperation and shared teleoperation rely on manipulation by an operator and visual feedback, the operator’s excessive workload and visual obstacles, such as flames, bubbles and debris, have made the application of robotic systems impractical in nuclear facility dismantling. Therefore, 3D imaging equipment capable of implementing the pre-planned path and target localization is key to the application of the semi-automated robotic system in nuclear facility dismantling.

Considering the radiation intensity of the dismantling site and the working space of the robotic system, it is necessary for the 3D imaging equipment to be able to measure up to 2.5 m away, to have a positioning accuracy within 1 mm, and to survive up to a cumulative dose of 1 kGy. During a survey of commercial underwater 3D scanners [5], Newton Labs’ NM200UW [6] was found to be usable in radioactive and underwater environments, but the NM200UW is insufficient for the requirements of a remote dismantling system in terms of a maximum measurable distance of 0.9 m and a radiation resistance of 43 Gy. Since Photoneo’s PhoXi XL [7] can measure up to 3.78 m and has a positional accuracy of 0.5 mm, we decided to develop 3D imaging equipment based on the PhoXi XL because it satisfies the requirements of the remote dismantling system. The PhoXi XL uses a continuous wave Laser Line Scanning (LLS) method and consists of a moving laser pointer made of a mirror galvanometer, a camera and an embedded computer for image processing.

We aimed to develop 3D imaging equipment based on the industrial 3D scanner PhoXi XL that is waterproof and has radiation resistance and refraction correction. Waterproofing could be solved simply by making a housing with windows and waterproof connectors. Radiation resistance could also be solved simply by mounting a shield on the front side since the main radiation source is the target structure on the front side in dismantling highly activated structures. However, 3D imaging equipment needs to have an optical path that can send and receive light to the front despite the front shielding. Refraction correction could be solved by refraction modeling, measurement experiments and parameter studies. Hidden technical details of the industrial 3D scanner cause many unknown parameters in the refraction model, so a process for optimizing unknown parameters through experimental results was necessary.

Radiation hardening and shielding are typical methods used for radiation protection. Radiation hardening makes electronic components and circuits radiation tolerant; shielding uses a solid or liquid material that absorbs radiation energy to block radiation. Radiation hardening can be a more fundamental solution compared to shielding and can minimize the increase in volume and weight, but radiation hardening can only be achieved by exchanging fragile electronic components with electronic components that are tolerable to radiation exposure. Therefore, shielding was the only choice for radiation protection of the 3D imaging equipment using an industrial 3D scanner, of which electronic components are not easily accessible to users.

To develop 3D imaging equipment, radiation hardening can be attempted. Zhao and Chi [8,9] developed an underwater 3D scanner using a continuous wave LLS method. To make the developed 3D scanners radiation tolerant, the electronic components and the camera need to be exchanged with radiation tolerant components [10,11], and the embedded computer for image processing needs to be located away from the radioactive environment. However, such an attempt may require a very long time and much effort to satisfy the requirements of the remote dismantling system. In a radioactive environment, the use of industrial products through shielding can be a very efficient alternative, as the study by Shin shows [12].

Refraction correction is required to improve the positional accuracy of the 3D point cloud measured in water, so that the position estimated by 3D registration represents the actual location of the scanned object. When a 3D scanner housed in waterproof housing scans an object in water through a window, the laser emitted from the laser pointer is refracted as it passes through the window and travels through the water, where it is reflected by the object and refracted into the camera through the window. Since the 3D scanner calculates the 3D position by triangulation principles, the angle changed by refraction causes a position error. From previous studies [8,9,13,14,15,16,17,18], all the parameters for refraction correction were known because all 3D scanners were designed and manufactured by the researchers themselves. In this study, unlike in previous studies, we optimized unknown parameters for refraction correction by an intuitive parameter study.

The goal of this study was to develop 3D imaging equipment for use with a robotic system that can accurately localize an activated target structure in water despite the position difference between the digital model and the actual target structure. The requirements of the 3D imaging equipment are the positional accuracy within 1 mm in a distance range of 0.5 to 3.5 m and radiation resistance of 1 kGy. To achieve the requirements, we designed a housing structure for radiation protection and waterproofing, calculating the shielding thickness which we verified through experiments, and performed refraction correction through refraction modeling, measurement experiments and parameter studies.

The following section presents the proposed housing design for radiation protection and waterproofing. Section 3 describes the process of investigating the initial radiation resistance, calculating the thickness of the shield, and verifying the shield thickness. Section 4 includes the process of refraction modeling for the developed 3D imaging equipment, performing measurement experiments in air and water, and studying unknown parameters of the refraction model. The conclusion section summarizes the study and states the potential contributions of the presented method.

## 2. Housing Design for Radiation Protection and Waterproof

A housing structure is designed to protect the industrial 3D scanner from water and radiation. The industrial 3d scanner that satisfies the requirements of the remote dismantling system is the XL model, which is a Photoneo’s Phoxi 3D scanner product [7]. However, due to the limited size of the laboratory, the industrial 3D scanner applied to this study was the L model, which has a slightly smaller scan volume than the XL model. The scanning range of the L model is 780 mm to 2150 mm, and the dimensions are 77 × 68 × 616 mm.

### 2.1. Optical Path Configuration

**Figure 1:**
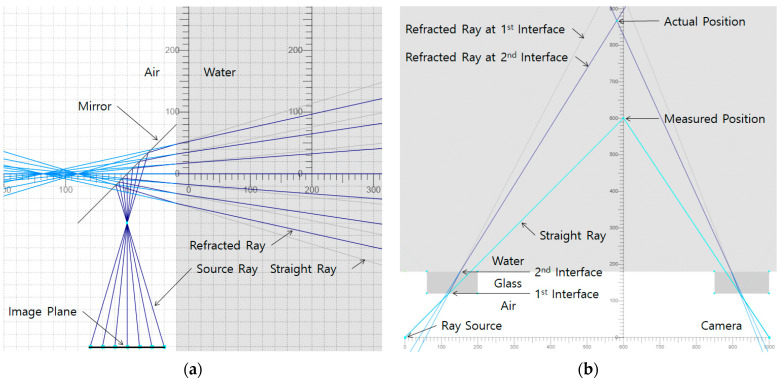
Ray Optics Simulation: (**a**) Vertical Plane; (**b**) Horizontal Plane.

The key to housing design is to protect the 3D scanner from radiation while not obstructing the view of the industrial 3D scanner. In this study, we used reflective mirrors so that the industrial 3D scanner could be protected from a front activated structure by a shield and be visually accessible to the outside. Ray optics simulations [19] were performed to schematically understand the phenomenon of seeing the outside underwater through a reflective mirror and a glass window. Figure 1a shows that the image projected onto the image plane through the reflective mirror is flipped and the refraction of the ray in water narrows the field of view. Figure 1b shows that when the 3D scanner measures a position in water, the measured position is closer than the actual position.

**Figure 2:**
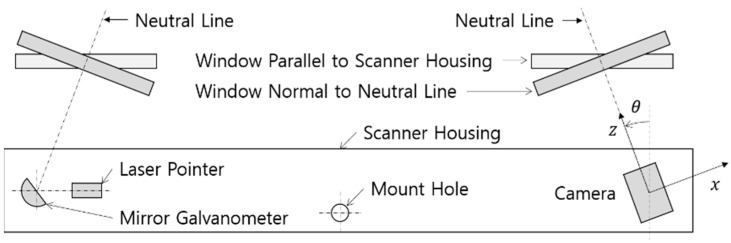
Optical system configuration of industrial 3D scanner.

The housing needed to be designed to minimize refraction because the magnitude of the refraction angle is proportional to the error of the calculated position due to a possible modeling error. Figure 2 shows two options for the window layout to configure the optical paths consisting of a camera, a laser pointer and a galvanometer. The neutral lines of the camera’s view and the laser projection pattern are inclined inward by angle θ. A window parallel to the scanner housing can contribute to size reduction, but the magnitude of the refraction angle increases, and the increased refraction angle causes larger errors. Therefore, a window normal to the camera’s z-axis was used.

**Figure 3:**
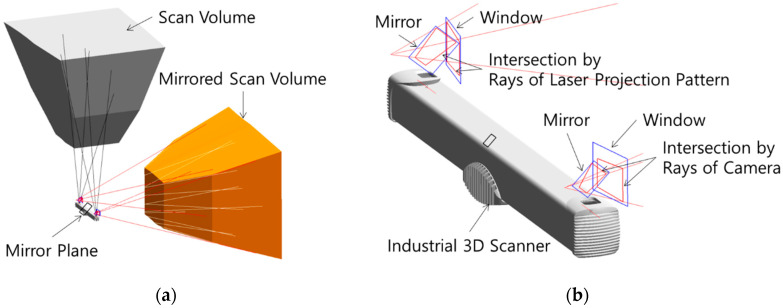
Optical Path Configuration: (**a**) Scan Volume; (**b**) Boundaries of Mirror and Window.

The optical path was configured as shown Figure 3. Figure 3a shows the scan volume and the mirrored scan volume of the industrial 3D scanner. Figure 3b shows the boundaries of the mirrors and windows, which surround respective intersections by planes and rays. Since the mirrored scan volume shown in Figure 3a is formed by one plane, the two mirror planes shown in Figure 3b belong to the same plane, but each has two boundaries to reduce the size of the mirror. Planes of windows are, respectively, normal to the mirrored neutral lines of the laser projection pattern and the camera’s view.

### 2.2. Housing Design

**Figure 4:**
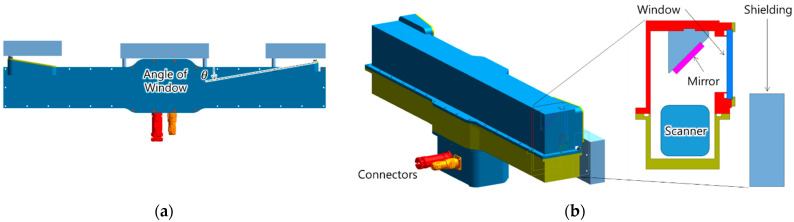
Housing Design: (**a**) Top View; (**b**) Isometric View.

The housing contains the industrial 3D scanner, connectors, first surface mirrors, windows and shielding as shown Figure 4. The industrial 3D scanner is fixed to the housing facing upwards and is provided with communication and power through waterproof connectors installed on the housing. The size and position of the two first surface mirrors are determined by the intersections as shown in Figure 3b. The windows located on the camera and the laser pointer are inclined inward by angle θ normal to the two neural lines as shown in the Figure 4a.

We designed the shielding to absorb gamma rays radiated from the activated target structure in front. To reduce the weight of 3D imaging equipment, the shielding was divided into three pieces and each shielding is placed in front of the laser pointer, camera and embedded computer for image processing as shown Figure 4a. At the actual dismantling workshop, the position of the industrial 3D scanner and the size of the shield can be changed.

## 3. Radiation Protection

Radiation protection through shielding is finally determined by calculating the thickness based on the initial radiation resistance, the sources of radiation and the shielding material. According to the radiological characterization of the reactor pressure vessel in a nuclear power plant, the main source emitting gamma rays is Co-60 [20,21]. Half value layers vary according to the energy of gamma rays and shield materials. When the source of radiation is Co-60 and the shield material is lead, half value layers is 1.2 cm [22]. Now we show the process of investigating the initial radiation resistance, calculating the shielding thickness and verifying final radiation resistance.

### 3.1. Initial Radiation Resistance

**Figure 5:**
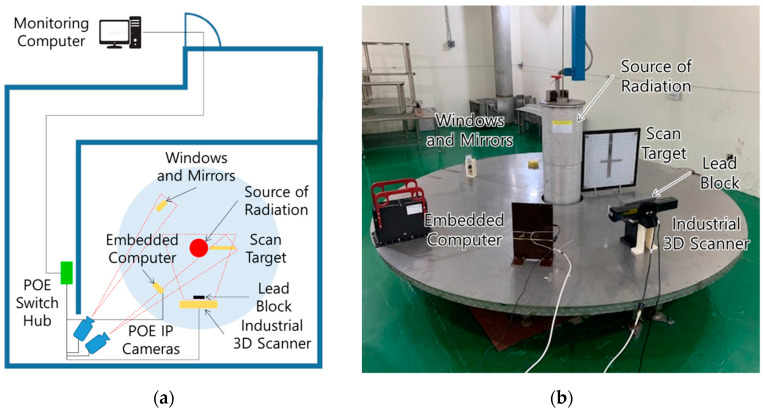
Irradiation Experimental Setup: (**a**) Layout Drawing; (**b**) Irradiation Facility.

An irradiation experiment was performed to investigate the initial radiation resistance of the industrial 3D scanner and to investigate changes in optical characteristics for window and mirror candidates, as shown in Figure 5. A thick lead block protects the embedded computer inside the industrial 3D scanner, because the embedded computer must survive to the end of the experiment to drive the laser pointer, mirror galvanometer and camera during the irradiation experiment. The initial radiation resistance of the embedded computer was investigated by an embedded computer of the same type. Window and mirror candidates are a fused silica window, an aluminum coated first surface mirror and a silver coated first surface mirror. One of the two IP cameras observes the scan target continuously. When an image change event is caused by the laser pattern projected by the industrial 3D scanner, the IP camera transmits the laser pattern image to the monitoring computer according to the image change event. The other IP camera observes the windows and mirrors and transmits images to the monitoring computer every 60 s. The monitoring computer requests a 3D point cloud from the industrial 3D scanner and requests a ping from the embedded computer every 60 s.

The source of radiation is Co-60 of which the energy spectrum is 1.17 and 1.33 MeV. The absorbed dose rate in the experiment is 4.32 × 10^1^ Gy/h, which is the dose rate at a location 730 mm away from the source of radiation. The total absorbed dose is 1.21 × 10^3^ Gy during the experiment and irradiation time is 28 h. The ambient laboratory temperature is 25.6 °C at the start of the experiment and 25.4 °C at the end of the experiment.

**Figure 6:**
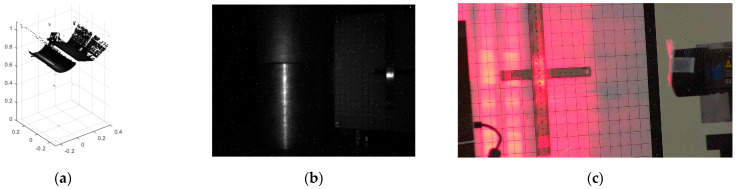
Experimental Data at 5H56M: (**a**) 3D Point Cloud; (**b**) Image of Camera; (**c**) Image of IP Camera.

The initial radiation resistance of the industrial 3D scanner is 259.4 Gy, since an abnormality occurred 5 h and 57 min after the start of the experiment. As shown in Figure 6, in the normal case, when the monitoring computer requests data from the industrial 3D scanner, the industrial 3D scanner sends the 3D point cloud and the image captured by the camera. At the same time, the IP camera detects an image change event caused by the laser pattern projected by the industrial 3D scanner and transmits the laser pattern image to the monitoring computer.

**Figure 7:**
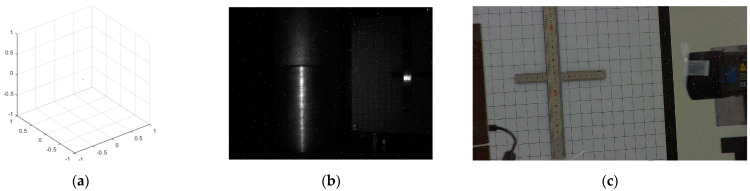
Experimental Data at 5H57M: (**a**) 3D Point Cloud; (**b**) Image of Camera; (**c**) Image of IP Camera.

At 5 h 57 min, as shown in Figure 7, when the monitoring computer requests data from the industrial 3D scanner, the image captured by the camera of the industrial 3D scanner is the same as the image in Figure 6b, but the 3D point cloud is almost empty. In Figure 7c, the reason that the IP camera transmits the image without any laser pattern is that the holding time of the laser pattern is shorter than normal, so when the IP camera detects the projected laser pattern and then sends the image to the monitoring computer, the laser pattern has already disappeared at that moment. The state shown in Figure 7 lasts for 5 min, after which the industrial 3D scanner stops responding. When the laser pattern completely stops working, the embedded computer also stops responding, and the IP camera stops sending images.

In the industrial 3D scanner, the most vulnerable component to radiation is the laser pattern projector, and the radiation resistances of the remaining components are unknown. Considering that the quality of the images captured by the camera in the industrial 3D scanner is maintained for the last 5 min, the radiation resistance of the camera is larger than the radiation resistance of the laser pattern projector. However, the exact radiation resistance of the camera cannot be determined. Because the monitoring program stops working due to no response of the industrial 3D scanner, the exact surviving time of the other embedded computer is unknown. At the ping test after the irradiation experiment, the embedded computer in the industrial 3D scanner responds normally and the other embedded computer without a shield does not respond.

**Figure 8:**
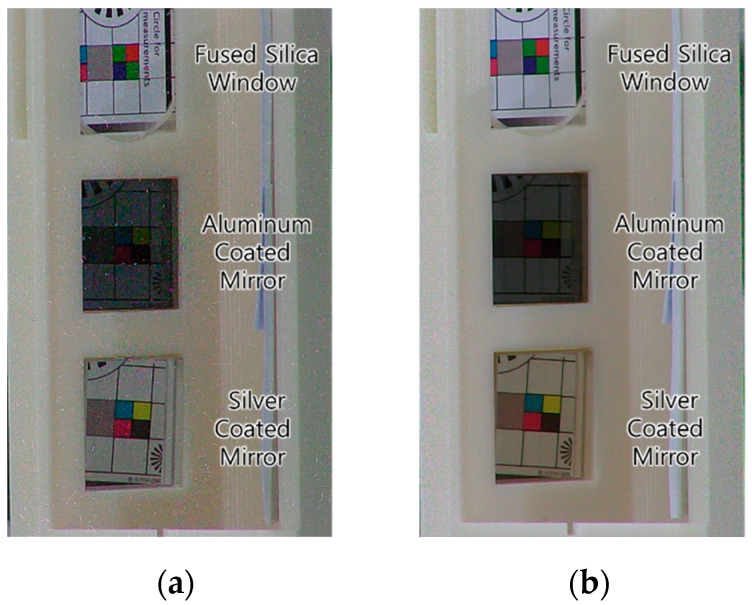
Optical Parts: (**a**) Initial State; (**b**) Final State.

Figure 8a is an image of the IP camera at the start of irradiation, and Figure 8b is an image of the IP camera at the end of irradiation. The noise observed in Figure 8a is the photon shot noise generated during irradiation, and it disappears at the end of irradiation as shown in Figure 8b. In all optical components, no significant deterioration is observed after irradiation with the naked eye. The reason why the silver-coated mirror in Figure 8b turned slightly yellow is because the substrate of the silver-coated mirror is discolored. In the case of a first surface mirror, the discoloration of the substrate does not affect the reflectivity of the mirror.

Table 1 shows the measurement results of light transmittance and light reflectance for irradiated components. In all optical components, no performance degradation of more than 1% is observed at 637 nm, the wavelength of the industrial 3D scanner’s laser. Therefore, all optical components are suitable for the 3D scanner in this study. Finally, the silver coated first surface mirror is selected because the reflectance of the silver coated first surface mirror is superior to that of the aluminum coated first surface mirror.

Since the degradation of optical components is less than 1% at 1.21 × 10^3^ Gy of the total absorbed dose, the radiation resistance of the developed 3D imaging equipment is estimated to be dominated by the shielding thickness.

### 3.2. Shielding Thickness Calculation

The shielding thickness is calculated by following the basic equation [21] that assumes a narrow beam of radiation penetrating a thin shield.

(1) $$X=X_{0}e^{-\frac{u}{\rho }\rho x}$$

where X is the exposure rate with the shield in place, X_0_ is the exposure rate without the shield, *u/ρ* is the mass attenuation coefficient, *ρ* is the density of the shielding material, and *x* is the thickness of the shield. If Equation (1) is used, since Co-60 emits 1173 and 1332 keV gamma rays, the exposure rate for the two gamma rays must be calculated and added. In this study, Half Value Layers (HVL) was used for simple calculations. A HVL is the thickness of material that reduces the radiation intensity by one-half.

(2) $$\mathrm{HVL}=\frac{0.693}{\mu }=\frac{0.693}{\left(\mu /\rho \right)\rho }$$

(3) $$X=X_{0}e^{-\frac{0.693}{\mathrm{HVL}}x}$$

(4) $$x=\frac{\mathrm{HVL}}{0.693}ln\frac{X_{0}}{X}$$

As shown in Table 2, when the shielding material is lead and the radiation source is Co-60, the HVL is 1.2 cm. The shielding thickness is calculated by Equation (4) where X_0_ is 1000 Gy and X is 259.4 Gy. The calculated shielding thickness is 3.0 cm, considering a margin, and the expected radiation resistance is 1467 Gy.

### 3.3. Radiation Resistance Verification

**Figure 9:**
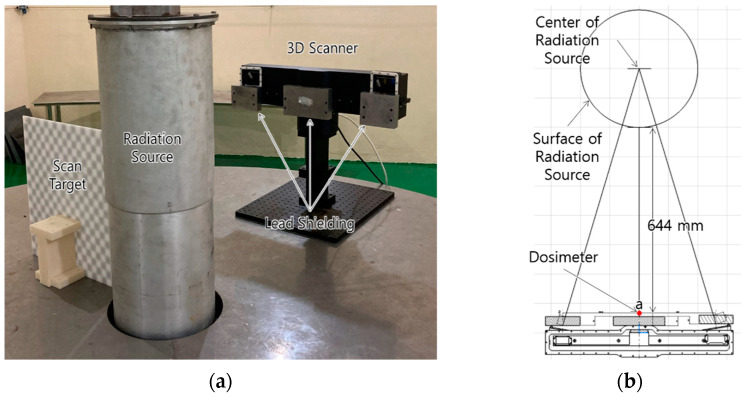
Verification Experiment: (**a**) Experimental Setup; (**b**) Detailed Layout Drawing.

An irradiation experiment was performed to verify the radiation resistance of the shielded 3D scanner, as shown in Figure 9. The industrial 3D scanner is housed in the housing manufactured in this study and it scans the target through mirrors and windows. The housing is equipped with three lead blocks with a 3 cm thickness. Three dosimeters are fixed in front of the components of the 3D scanner, as shown in a, b, and c in Figure 9b. Dosimeter a is fixed in front of the shielding at the position closest to the radiation source, and dosimeters b and c are fixed behind the shielding at the position of the camera and laser pattern projector, respectively. Dosimeter a is measured at a distance of 644 mm from the surface of the radiation source. The irradiation experiment is performed for 24 h at a dose rate of 64.3 Gy/h based on dosimeter a.

**Figure 10:**
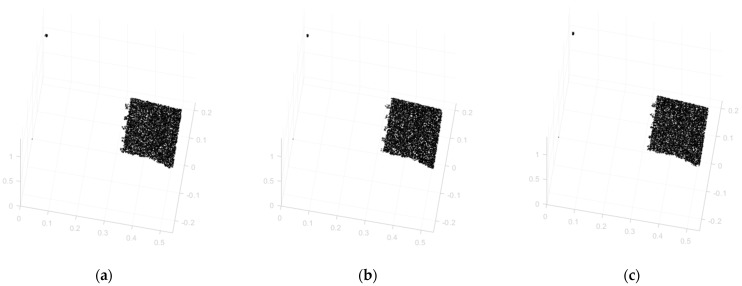
Measured 3D Point Cloud: (**a**) At Start; (**b**) Before Abnormal Termination; (**c**) At End.

**Figure 11:**
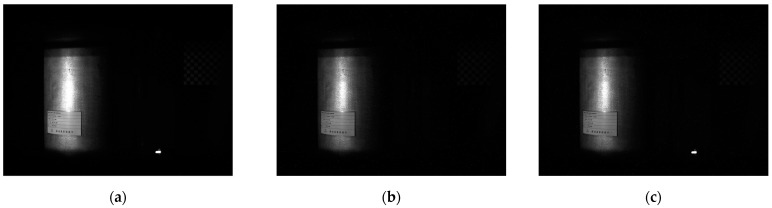
Images of Scanner: (**a**) At Start; (**b**) Before Abnormal Termination; (**c**) At End.

After the irradiation experiment for 24 h the 3D scanner operated normally, and the total absorbed dose was 1534.4 Gy based on dosimeter a. An abnormal termination occurred at 22 h and 34 min during the irradiation experiment. However, the abnormal termination turned out to be temporary because restarting the monitoring process created no problems. Figure 10 and Figure 11 show the 3D point clouds and images captured by the industrial 3D scanner at the start of the experiment (a), just before the abnormal termination (b), and at the end of the experiment (c). No degradation of quality is observed for any of the 3D point clouds and images. In Figure 10, since the cylinder-shaped surface of the radiation source is closer than 800 mm from the 3D imaging equipment, only the scan target is measured.

**Figure 12:**
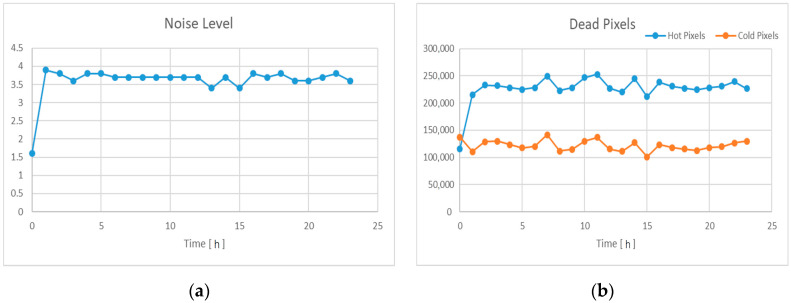
Image Quality Analysis: (**a**) Noise Level; (**b**) Dead Pixels.

Figure 12 shows the results from analyzing images captured by the 3D scanner using the image analysis tool. In the 3D scanner based on the LLS method, the image quality has a decisive effect on the quality of the measured 3D point cloud, so the result of the image quality analysis may represent the quality of the measured 3D point cloud. The noise level and hot pixels increase rapidly after irradiation starts, but do not show a distinct increase trend during 24 h of the experiment. Since position estimation based on 3D registration uses hundreds or more points, such random noise may not significantly affect position accuracy. Therefore, the developed 3D imaging equipment is expected to be successfully applied to the remote dismantling system in nuclear facility decommissioning.

The total absorbed dose of dosimeter b fixed in front of the laser pattern projector is 184.2 Gy, and the total absorbed dose of dosimeter c fixed in front of the camera is 191.8 Gy. When calculating the actual penetration thickness of gamma rays based on the drawing in Figure 9b, the shielding thickness is 31.3 mm and the total absorbed dose without shielding is calculated as 1112.3 Gy and 1171.6 Gy in dosimeter b and dosimeter c, respectively. In a previous experiment the laser pattern projector was investigated and found to be the weakest to radiation, and in this verification experiment the total absorbed dose of the laser pattern projector equipped with shielding is 1112.3 Gy, so we considered this radiation protection as successful. In addition, considering the radiation resistance of the applied optical components and the result of image quality analysis, easy improvement of the radiation resistance is expected through shielding design reinforcement.

## 4. Refraction Correction

Refraction correction finds the light plane and camera ray from the 3D coordinates of the point measured by the industrial 3D scanner, and then finds the corrected 3D coordinates of the point by recalculating the refracted light plane and the refracted camera ray based on the refraction model. The light plane is the equation of the plane projected by the laser pointer, and the camera ray is the equation of the line connecting the point on the camera’s image plane and the point measured by the industrial 3D scanner. An accurate refraction model is required for this calculation. However, the exact values required for the refraction model are rarely found due to the inaccessibility of design information in the industrial 3D scanner. In this study, we developed a basic refraction model and studied the unknown parameters by a simple intuitive approach.

### 4.1. Refraction Modeling

**Figure 13:**
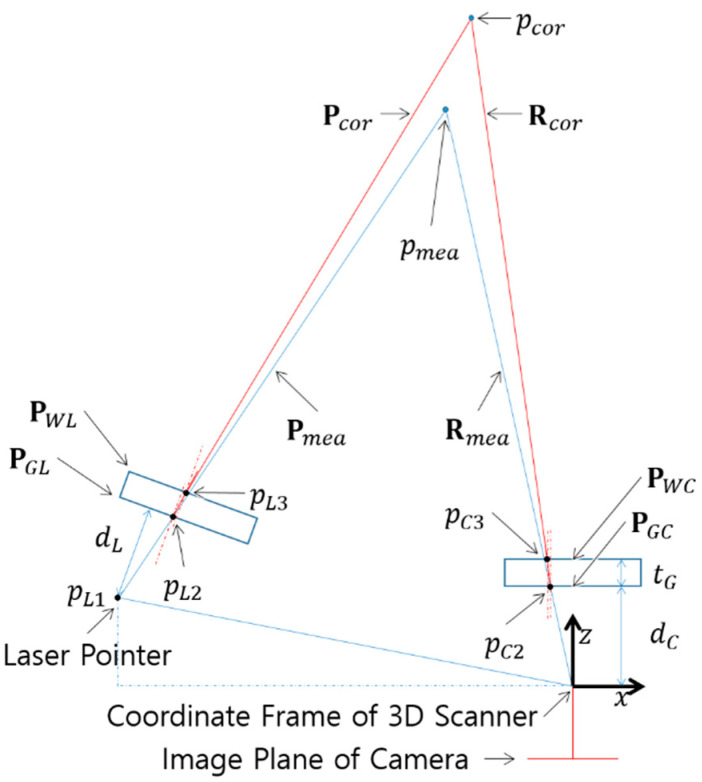
Refraction Model.

A refraction model is developed fundamentally based on a pinhole model and a perspective projection [23]. Although the calculation algorithm performed inside the industrial 3D scanner is unknown, the point measured by the industrial 3D scanner can be reconstructed with a light plane and camera ray, assuming that the positions of the laser pointer and camera are known. Figure 13 shows the reconstructed light plane and camera ray for the measured point *p_mea_* and the refraction correction point *p_cor_* on the x-z plane of the coordinate frame, which is located in the pinhole of the 3D scanner’s camera. In front of the laser pointer and camera, a glass window is installed at distances of *d_L_* and *d_C_*, respectively. The thickness of both glass windows is the same at *t_G_*. On both planes of the glass window positioned at the laser pointer, the plane **P***_GL_* is the interface between air and glass, and the plane **P***_WL_* is the interface between glass and water. Likewise, the plane **P***_GC_* is the interface between air and glass, and the plane **P***_WC_* is the interface between glass and water at the glass window positioned at the camera.

**Figure 14:**
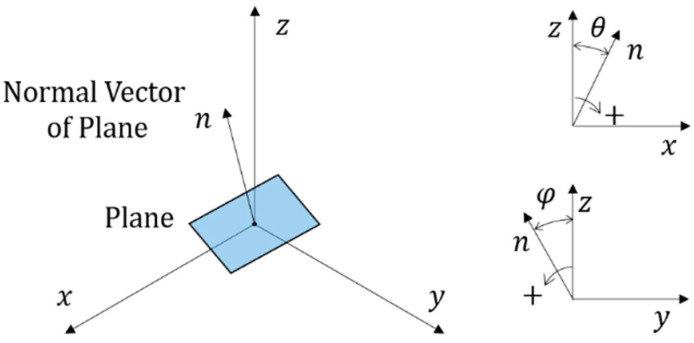
Plane Model.

The plane of the glass window located in 3D space can be represented as in Figure 14 for the angles *θ* and *φ*, at which the normal vector is inclined with respect to the z-axis of the coordinate frame.

The planes **P***_GC_*, **P***_WC_*, **P***_GL_* and **P***_WL_* are given by Equations (5)–(8)

(5) $$P_{GC}={\left\{p\;:n^{t}\left(p-q_{P}\right)=0\right\}}_{GC}\;where\;n=\left(\begin{array}{ccc} \tan\theta_{C} & \tan\varphi_{C} & 1 \end{array}\right),\;q_{P}=\left(\begin{array}{ccc} 0 & 0 & \sqrt{\tan^{2}\theta_{C}+\tan^{2}\varphi_{C}+1}\cdot d_{C} \end{array}\right)$$

(6) $$P_{WC}={\left\{p\;:n^{t}\left(p-q_{P}\right)=0\right\}}_{WC}\;where\;n=\left(\begin{array}{ccc} \tan\theta_{C} & \tan\varphi_{C} & 1 \end{array}\right),\;q_{P}=\left(\begin{array}{ccc} 0 & 0 & \sqrt{\tan^{2}\theta_{C}+\tan^{2}\varphi_{C}+1}\cdot \left(d_{C}+t_{G}\right) \end{array}\right)$$

(7) $$P_{GL}={\left\{p\;:n^{t}\left(p-q_{P}\right)=0\right\}}_{GL}\;where\;n=\left(\begin{array}{ccc} \tan\theta_{L} & \tan\varphi_{L} & 1 \end{array}\right),\;q_{P}=\left(\begin{array}{ccc} 0 & 0 & \sqrt{\tan^{2}\theta_{L}+\tan^{2}\varphi_{L}+1}\cdot d_{L}-\tan\theta_{L}x_{L}-\tan\varphi_{L}y_{L}-z_{L} \end{array}\right)$$

(8) $$P_{WL}={\left\{p\;:n^{t}\left(p-q_{P}\right)=0\right\}}_{WL}\;where\;n=\left(\begin{array}{ccc} \tan\theta_{L} & \tan\varphi_{L} & 1 \end{array}\right),\;q_{P}=\left(\begin{array}{ccc} 0 & 0 & \sqrt{\tan^{2}\theta_{L}+\tan^{2}\varphi_{L}+1}\cdot \left(d_{L}+t_{G}\right)-\tan\theta_{L}x_{L}-\tan\varphi_{L}y_{L}-z_{L} \end{array}\right)$$

where *θ***_C_** and *φ_C_* are the angles of the glass window positioned at the camera, *θ_L_* and *φ_L_* are the angles of the glass window positioned at the laser, and the point (*x_L_*, *y_L_*, *z_L_*) represents *p_L_*_1_ of the laser.

When *p_L_*_1_, *θ_C_*, *φ_C_*, *θ_L_*, *φ_L_*, *d_C_*, *d_L_*, *t_G_* and *p_mea_* are known, the refraction correction point *p_cor_* can be derived by Equations (9)–(28). First, the camera ray **R***_mea_* are given by Equation (9)

(9) $$R_{mea}={\left\{p=q_{L}+\lambda v:\;\lambda \geq 0\right\}}_{mea}\;where\;q_{L}=0,\;v=v_{C1}=p_{mea}$$

The intersection point *p_C2_* of **R***_mea_* and **P***_GC_* can be computed by Equations (10) and (11).

(10) $$n_{GC}^{t}\left(p-q_{GC}\right)=n^{t}\left(\lambda_{C2}v_{C1}+q_{L}-q_{GC}\right),\;\lambda_{C2}=\frac{n_{GC}^{t}\left(q_{GC}-q_{L}\right)}{n_{GC}^{t}v_{C1}}\;\mathrm{where}\;n_{GC}=\left(\begin{array}{ccc} \tan\theta_{C} & \tan\varphi_{C} & 1 \end{array}\right),\;q_{GC}=\left(\begin{array}{ccc} 0 & 0 & \sqrt{\tan^{2}\theta_{C}+\tan^{2}\varphi_{C}+1}\cdot d_{C} \end{array}\right),\;q_{L}=0,\;v_{C1}=p_{mea}$$

(11) $$p_{C2}=\lambda_{C2}v_{C1}$$

**Figure 15:**
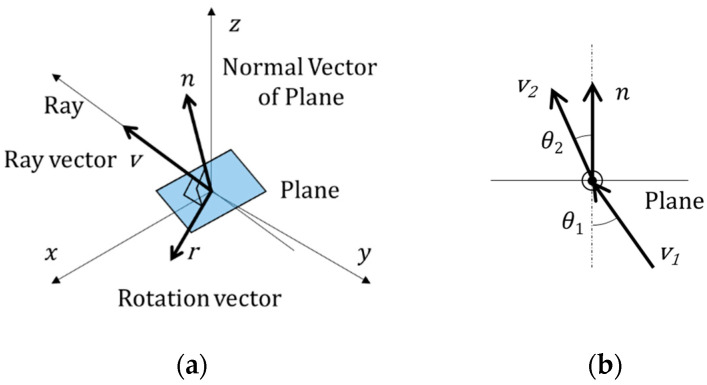
Refraction of a ray: (**a**) Isometric view; (**b**) Plane normal to the rotation vector.

The vector *v*_2_ of refraction with the incident vector *v*_1_ and the interface normal *n* is computed by Snell’s law, quaternion rotation operation and Equations (12)–(17) as shown Figure 15.

(12) $$\theta_{1}=\cos^{-1}\frac{v_{1}\cdot n}{\left\|v_{1}\|\|n\right\|}$$

(13) $$\theta_{2}=\sin^{-1}\frac{N_{1}\sin\theta_{1}}{N_{2}}\;where\;N_{1}\;and\;N_{2}\;are\;refractive\;indice$$

(14) $$r=\frac{v_{1}\times n}{\left\|v_{1}\|\|n\right\|}=\left(\begin{array}{ccc} r_{x} & r_{y} & r_{z} \end{array}\right)$$

(15) $$q=Q\left(v_{1},\;n,N_{1},N_{2}\right)=\left[\begin{array}{cc} \cos\frac{\theta_{2}}{2} & \sin\frac{\theta_{2}}{2}\left(\begin{array}{ccc} r_{x}i & r_{y}j & r_{z}k \end{array}\right) \end{array}\right]$$

(16) $$n=\left[\begin{array}{cc} 0 & n \end{array}\right]=\left[\begin{array}{cc} 0 & \begin{array}{ccc} n_{x}i & n_{y}j & n_{z}k \end{array} \end{array}\right]$$

(17) $$v_{2}=qnq^{*}\;where\;v_{2}=\left[\begin{array}{cc} 0 & v_{2} \end{array}\right]=\left[\begin{array}{cc} 0 & \begin{array}{ccc} v_{2x}i & v_{2y}j & v_{2z}k \end{array} \end{array}\right]$$

Therefore, the refracted camera ray **R***_C_*_2_ at the point *p_C_*_2_ on the plane **P***_GC_* is computed by Equations (18) and (19).

(18) $$v_{C2}=qn_{GC}q^{*},\;where\;n_{GC}=\left[\begin{array}{cc} 0 & n_{GC} \end{array}\right],\;q=Q\left(v_{C1},n_{GC},N_{air},N_{glass}\right)$$

(19) $$R_{C2}={\left\{p=q_{L}+\lambda v:\;\lambda \geq 0\right\}}_{C2},\;where\;q_{L}=p_{C2},\;v=v_{C2}$$

The intersection point *p_C3_* of **R***_C_*_2_ and **P***_WC_* is computed by Equation (20).

(20) $$p_{C3}=\lambda_{C3}v_{C2},\;where,\;\lambda_{C3}=\frac{n_{WC}^{t}\left(q_{WC}-p_{C2}\right)}{n_{WC}^{t}v_{C2}}$$

In the same way, the corrected camera ray **R***_cor_* at the point *p_C_*_3_ on the plane **P***_WC_* is computed by following Equations (21) and (22).

(21) $$v_{C3}=qn_{WC}q^{*}\;where\;n_{WC}=\left[\begin{array}{cc} 0 & n_{WC} \end{array}\right],\;q=Q\left(v_{C2},n_{WC},N_{glass},N_{water}\right)$$

(22) $$R_{cor}={\left\{p=q_{L}+\lambda v:\;\lambda \geq 0\right\}}_{cor}\;where\;q_{L}=p_{C3},\;v=v_{C3}$$

The corrected *p_cor_* is the intersection of the corrected camera ray **R***_cor_* and the corrected light plane **P***_cor_*, so the corrected light plane **P***_cor_* needed to be found. Since the laser starts from a point light source, the laser rays **R***_L_*_1_, **R***_L_*_2_ and **R***_L_*_3_ are calculated in the same way as the previous camera ray calculation method, and the corrected light plane **P***_cor_* can be found with the condition that it is perpendicular to the xz plane and includes the finally refracted laser ray **R***_L_*_3_. The corrected light plane **P***_cor_* is computed by following Equations (23)–(30).

(23) $$R_{L1}={\left\{p=q_{L}+\lambda v:\;\lambda \geq 0\right\}}_{L1}\;where\;q_{L}=p_{L1},\;v=v_{L1}=p_{mea}-p_{L1}$$

(24) $$p_{L2}=p_{L1}+\lambda_{L2}v_{L1}\;where\;\lambda_{L2}=\frac{n_{GL}^{t}\left(q_{GL}-p_{L1}\right)}{n_{GL}^{t}v_{L1}}$$

(25) $$v_{L2}=qn_{GL}q^{*}\;where\;n_{GL}=\left[\begin{array}{cc} 0 & n_{GL} \end{array}\right],\;q=Q\left(v_{L1},n_{GL},N_{air},N_{glass}\right)$$

(26) $$R_{L2}={\left\{p=q_{L}+\lambda v:\;\lambda \geq 0\right\}}_{L2}\;where\;q_{L}=p_{L2},\;v=v_{L2}$$

(27) $$p_{L3}=p_{L2}+\lambda_{L3}v_{L2}\;where\;\lambda_{L3}=\frac{n_{WL}^{t}\left(q_{WL}-p_{L2}\right)}{n_{WL}^{t}v_{L2}}$$

(28) $$v_{L3}=qn_{WL}q^{*}\;where\;n_{WL}=\left[\begin{array}{cc} 0 & n_{WL} \end{array}\right],\;q=Q\left(v_{L2},n_{WL},N_{glass},N_{water}\right)$$

(29) $$R_{L3}={\left\{p=q_{L}+\lambda v:\;\lambda \geq 0\right\}}_{L3}\;where\;q_{L}=p_{L3},\;v=v_{L3}$$

(30) $$P_{cor}={\left\{p\;:n^{t}\left(p-q_{P}\right)=0\right\}}_{cor}\;where\;n=\left(\begin{array}{ccc} -v_{L3z} & 0 & v_{L3x} \end{array}\right),\;q_{P}=p_{L3}$$

Finally, the corrected *p_cor_* is found by computing the intersection of the corrected camera ray **R***_cor_* and the corrected light plane **P***_cor_* as following Equation (31).

(31) $$p_{cor}=p_{C3}+\lambda_{cor}v_{C3}\;where\;\lambda_{cor}=\frac{n_{cor}^{t}\left(p_{L3}-p_{C3}\right)}{n_{cor}^{t}v_{C3}}$$

### 4.2. Experiments and Results

**Figure 16:**
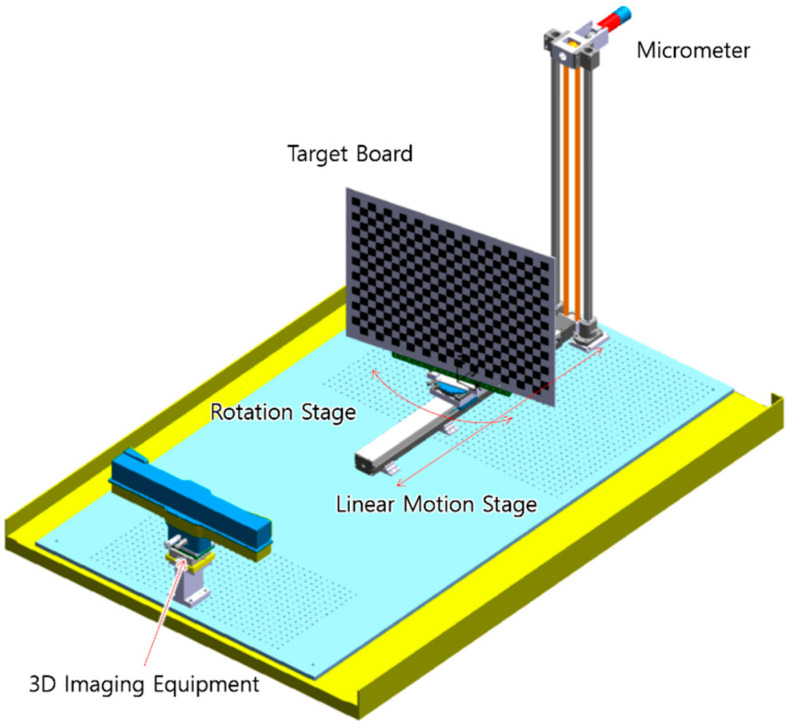
Design of experimental equipment.

To find unknown parameters in the presented refraction model, experiments to measure 3D point clouds of a target board were performed in air and water at the same distance. The experimental equipment consisted of a water tank, a 3D scanner, a linear motion stage, a rotation stage, a micrometer, and a target board as shown Figure 16. At the bottom of the water tank, a bread board with an M6 threaded hole pattern was installed to fix components in the correct position. The 3D scanner was housed in the manufactured waterproof housing and fixed to one end of the breadboard. The linear motion stage was fixed longitudinally to vary the distance between the 3D scanner and the target board. The rotation stage was fixed on the movable table of the linear motion stage and aligned the target board parallel to the image plane of the 3D scanner. The micrometer was connected to the drive shaft of the linear motion stage by a timing belt, so that the position of the movable table could be precisely adjusted. The target board was a plate with a 30 mm size checkered pattern printed on an 800 × 600 mm aluminum plate.

**Figure 17:**
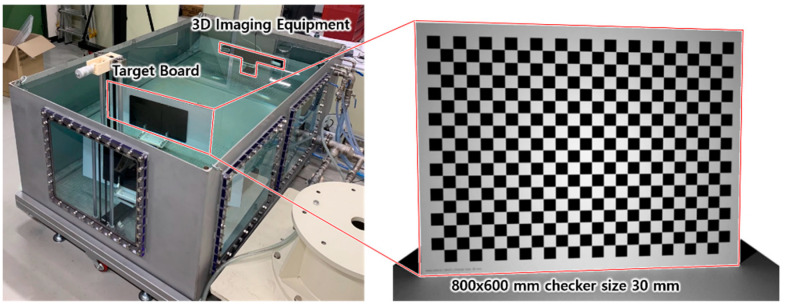
Experimental setup.

Figure 17 shows the experimental setup with the water tank filled and the target board.

**Figure 18:**
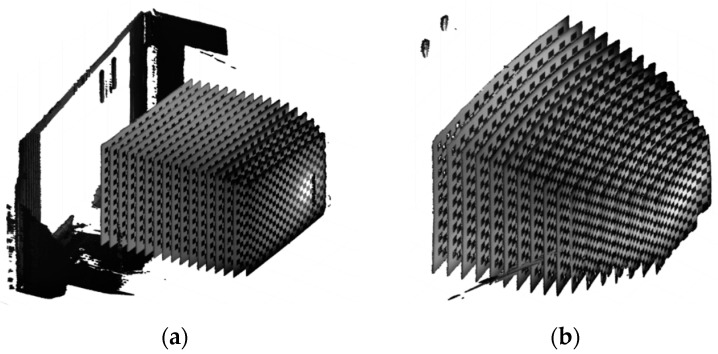
Obtained 3d point cloud: (**a**) in air; (**b**) in water.

Figure 18 shows the results of 17 scans in air (a), and water (b), while the movable table moves from 0 mm to 800 mm at 50 mm intervals. The 3D point clouds obtained in water are enlarged and curved compared to 3D point clouds obtained in air.

**Figure 19:**
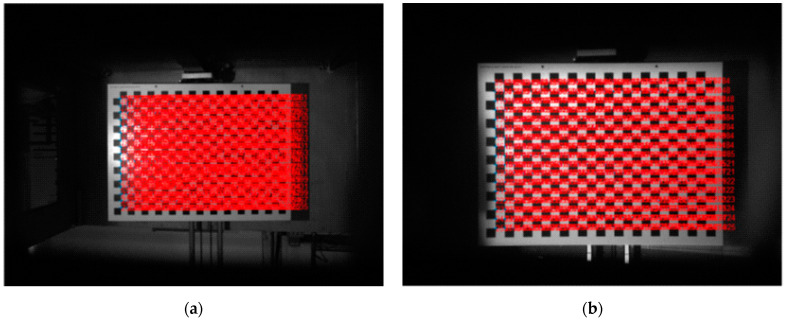
Results of checkerboard detection: (**a**) air; (**b**) water.

**Figure 20:**
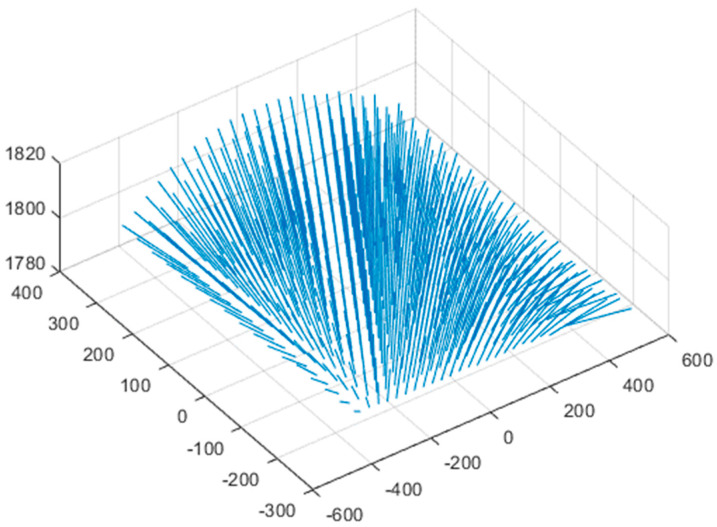
Estimation of measurement error due to refraction.

Figure 19 shows the image captured by the camera of the industrial 3D scanner, and the results of checkerboard detection at the position where the micrometer indicates 0 mm. Since the indexes of the image and the 3D point cloud are the same, the 3D coordinates of the points found by checkerboard detection in the image can be found. Therefore, we could find a pair of 3D points pointing to the same position in the 3D point cloud measured in water and air, and the pair of 3D points could be used to find the error of the point measured underwater. For example, the 3D point indicated by the upper right dot in the image of Figure 19a should have the same position as the 3D point indicated by the upper right dot in the image of Figure 19b. If the positions of the two 3D points are different, the difference in positions indicates a measurement error due to refraction. Figure 20 shows 408 pairs and measurement errors estimated from Figure 19.

**Figure 21:**
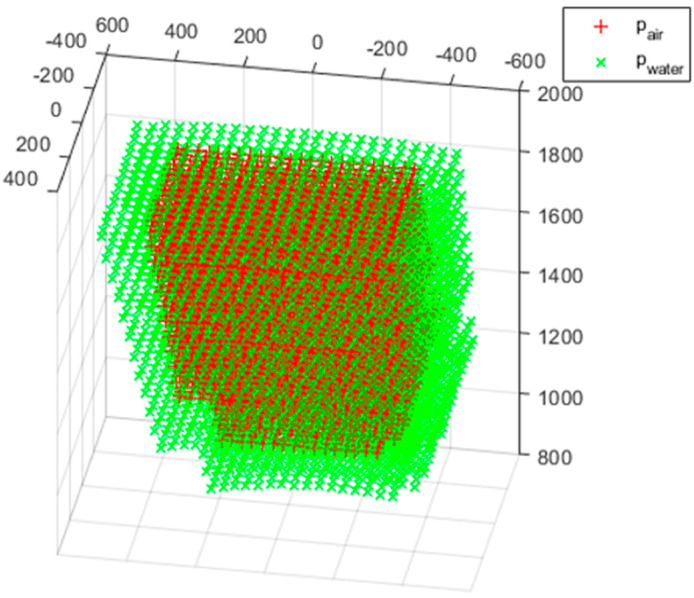
A pair of measurement error.

A pair of measurement errors was obtained by processing and merging 15 pairs of 3D point clouds with the previously described method. The pair of measurement errors consists of points pair measured in air and points pwater measured in water as shown Figure 21. The 16th and 17th measured 3D point clouds were excluded because the number of measured point clouds was small, and the measured 3D point clouds deviate from the proper measurement range because of narrowing the angle of view and appearing closer than the actual position of the object in water.

### 4.3. Parameter Study and Refraction Correction Result

In the refraction model presented in this study, the variables to find the refraction correction point *p_cor_* are *p_L1_*, *θ_C_*, *φ_C_*, *θ_L_*, *φ_L_*, *d_C_*, *d_L_*, *t_G_* and *p_mea_*. Among the listed variables, the glass thickness *t_G_* can be considered as a constant and *p_mea_* is a value measured by the 3D scanner, so *p_L1x_*, *p_L1y_*, *p_L1z_*, *θ_C_*, *φ_C_*, *θ_L_*, *φ_L_*, *d_C_* and *d_L_* can be considered as unknown parameters. The measured point *p_mea_* is the same as the points *p_water_* measured in water.

The objective function is the sum of refraction correction errors, which are the distances between the refraction correction points *p_cor_* and the points *p_air_* measured in air, and the goal of the parameter study was to minimize the objective function. Equation (32) represents the objective function *J*.

(32) $$J=\overset{n}{\underset{i=1}{\sum }}\|p_{cor}-p_{air}\|,\;where\;p_{cor}=f\left(\theta_{C},\varphi_{C},\theta_{L},\varphi_{L},d_{C},d_{L},p_{L1x},\;p_{L1y},p_{L1z}\right)$$

In this study, we attempted a parameter study with an intuitive and simple approach rather than a sophisticated theoretical approach. A discrete linear vector space was generated for each parameter, and parameter values that minimize the objective function were selected in the generated vector space. However, when the objective function simultaneously searched for the vector space of all the nine parameters, the number of cases increased exponentially, and the search time was too long. Therefore, at the beginning of the search, a strategy of combining parameters with similar characteristics and searching was attempted, and in the stationary phase of the search, a strategy of generating a random combination for the entire vector space and searching of all parameters was attempted. The search by a random combination made a homogeneous search possible for the entire vector space and reduction in search time through parallel operation, despite the incomplete search of the entire vector space.

**Figure 22:**
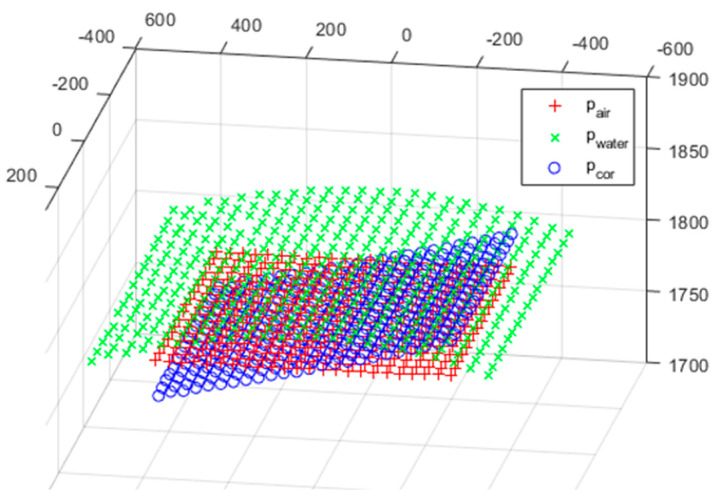
Initial error of refraction correction.

The initial values of all parameters were obtained from the CAD geometry of the housing design in Section 2.2, and the values are shown in Table 3. The mean error calculated from the initial values is 12.2 mm, and the 95% error is 101.2 mm and the 99% error is 138.0 mm in the normal distribution of the error. The reason that 99% error should be considered is that the error guaranteed by the 3D scanner presented in this study needs to be verified. Considering the result of refraction correction based on the initial value, the 99% error reveals that the characteristics of refraction correction are very bad, and Figure 22 shows the characteristics well. Figure 22 is the initial error of refraction correction using the first pair of 3D point cloud measured in air and water, which shows that the plane of the refraction correction points *p_cor_* is inclined in relation to the plane of the points *p_air_* measured in the air.

Table 3 shows the results of the parameter study by the search strategy described above. The decrease in mean error is not evident in the case of No. 1, in which the search to minimize the objective function is performed in the vector space of *θ_C_* and *φ_C_*_,_ and No. 2, in which the search is performed in the vector space of *θ_L_* and *φ_L_*. A distinct decrease in mean error is seen in case 3 searching on the vector space of *d_C_* and *d_L_*. Through cases 4, 5, and 6, the mean error is reduced to 0.57 mm to reach the precision required by the remote dismantling system described above. In particular, as the difference between the 99% error and the mean error is reduced, the error distribution is considerably improved. A homogeneous 3D registration error can be expected in the entire scan volume.

**Figure 23:**
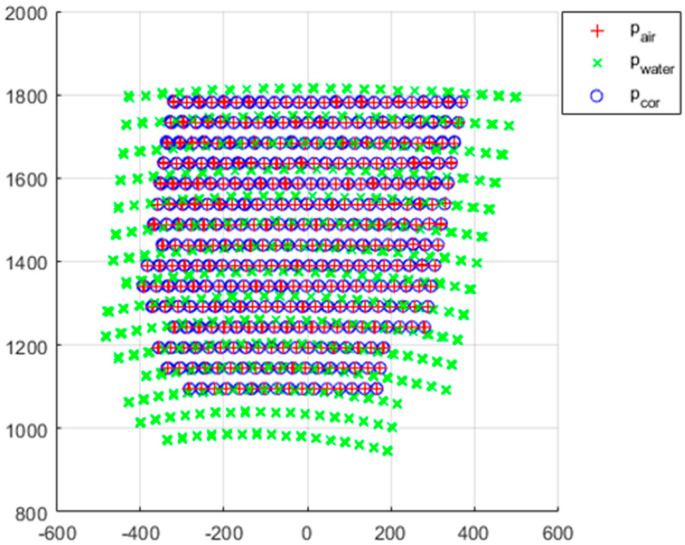
Refraction correction result.

Figure 23 shows the results of the refractive correction performed on the entire scan volume. The *p_cor_* with the *p_water_* transformed through refraction correction appears to be visually the same as *p_air_*.

**Figure 24:**
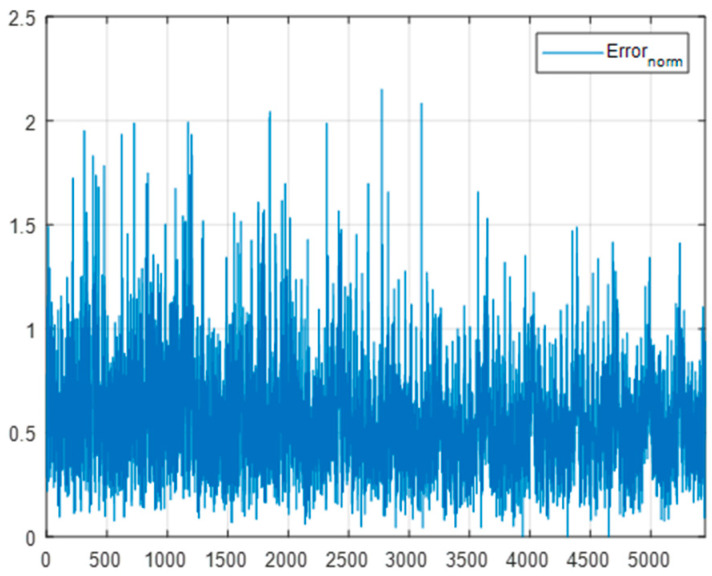
Measurement error.

Figure 24 shows the measurement error for all points. Many peak errors of 1 mm or more are observed, exceeding the mean error and 99% error. However, such peak errors may not significantly affect the 3D registration method for finding the position of the target object through a 3D point cloud consisting of hundreds of points.

## 5. Conclusions

3D imaging equipment required by the remote decommissioning system in the field of nuclear power plant decommissioning was successfully developed using an industrial 3D scanner. The 3D imaging equipment can survive up to a cumulative dose of 1 kGy and measure a 3D point cloud in air and in water. The measured 3D point cloud is accurate enough to estimate the position of a target object with an error of less than 1 mm. To achieve the goal, we selected a suitable industrial 3D scanner, designed a housing structure for water and radiation protection, and provided radiation protection through shielding and accuracy improvement through refraction correction. We proved the validity of the optical path configuration using mirrors, the shielding thickness calculation method, and the refraction modeling equations through the experimental results. The proposed method is expected to contribute to various applications requiring 3D imaging equipment in radioactive or underwater environments.

## Figures and Tables

**Table 1:** Light Transmittance for Window and Light Reflectance for Mirror.

Optical Parts	Wavelength (nm)	Normal Parts (%)	Irradiated Parts (%)	Results
Fused Silica Window	400	90.1	88.3	−1.8%
	700	97.3	96.6	−0.7%
Aluminum Coated First Surface Mirror	400	91.1	90.9	−0.2%
	700	80.5	79.9	−0.6%
Silver Coated First Surface Mirror	400	96	95.1	−0.9%
	700	98	97.5	−0.5%

**Table 2:** Approximate Half Value Layers in cm.

Energy (MeV)	Uranium	Tungsten	Lead	Iron	Concrete	Water
0.5			0.51	1.0	3.3	7.62
1.0			0.76	1.52	4.57	9.91
1.5			1.27	1.78	5.84	12.19
2.0			1.52	2.03	6.6	13.97
Ir-192	0.28	0.33	0.48	1.27	4.5	
Cs-137			0.65	1.6	4.8	
Co-60	0.69	0.79	1.2	2.1	6.2	
Ra-226			1.66	2.2	6.9	

**Table 3:** Parameter study.

No.	Mean Error (mm)	95% Error (mm)	99% Error (mm)	$\begin{array}{c} \theta_{C}\\ \left(\mathrm{Deg}.\right) \end{array}$	$\begin{array}{c} \varphi_{C}\\ \left(\mathrm{Deg}.\right) \end{array}$	$\begin{array}{c} \theta_{L}\\ \left(\mathrm{Deg}.\right) \end{array}$	$\begin{array}{c} \varphi_{L}\\ \left(\mathrm{Deg}.\right) \end{array}$	*d*_*C*_ (mm)	*d*_*L*_ (mm)	$\begin{array}{c} p_{L1x}\\ \left(\mathrm{mm}\right) \end{array}$	$\begin{array}{c} p_{L1y}\\ \left(\mathrm{mm}\right) \end{array}$	$\begin{array}{c} p_{L1z}\\ \left(\mathrm{mm}\right) \end{array}$
Init.	12.1812	101.1734	138.0446	0	0	18.8	0	69.4	79	−542.5	−8.7	87.1
1	12.0995	95.6218	130.2267	0.07	−0.12	18.8	0	69.4	79	−542.5	−8.7	87.1
2	11.6205	93.4643	127.3738	0.07	−0.12	18.57	0.24	69.4	79	−542.5	−8.7	87.1
3	2.015	2.9558	3.3455	0	−0.05	18.69	0.5	89.4	0	−542.5	−8.7	87.1
4	1.5879	1.8913	2.0171	0	−0.05	18.69	0.5	89.4	0	−537.5	−27.7	97
5	0.8352	1.0931	1.1999	−0.22	−0.09	18.485	1.18	89.4	0	−537.5	−27.7	97
6	0.5683	0.6895	0.7397	−0.0038	−0.0016	0.3226	0.0206	82.6	0	−536.3	−34.8	100.2
7	0.5531	0.6728	0.7222	−0.225	−0.1025	18.61	1.155	81.8	0	−539.1	−32.2	103.6

## Data Availability

Not applicable.
